# Effectiveness of a self-management program for dual sensory impaired seniors in aged care settings: study protocol for a cluster randomized controlled trial

**DOI:** 10.1186/1745-6215-14-321

**Published:** 2013-10-07

**Authors:** Lieve M Roets-Merken, Maud JL Graff, Sytse U Zuidema, Pieter GJM Hermsen, Steven Teerenstra, Gertrudis IJM Kempen, Myrra JFJ Vernooij-Dassen

**Affiliations:** 1 Scientific Institute for Quality of Healthcare, Radboud University Nijmegen Medical Centre, Geert Grooteplein-Zuid 21, Nijmegen 6525 EZ, The Netherlands; 2 Centre of Evidence-Based Practice, Department of Rehabilitation, Radboud University Nijmegen Medical Centre, Geert Grooteplein-Zuid 10, Nijmegen 6525 GA, The Netherlands; 3 Department of General Practice, University of Groningen, University Medical Centre Groningen, Hanzeplein 1, Groningen 9700 RB, The Netherlands; 4 Maasduinen Foundation, Vredesplein 100, Waalwijk 5142 RT, The Netherlands; 5 Department for Health Evidence, Biostatistics Section, Radboud University Nijmegen Medical Centre, Geert Grooteplein-Zuid 10, Nijmegen 6525 GA, The Netherlands; 6 CAPHRI School for Public Health and Primary Care, Faculty of Health, Medicine and Life Sciences, Department of Health Services Research, Maastricht University, Duboisdomein 30, Maastricht 6229 GT, The Netherlands; 7 Kalorama Foundation, Nieuwe Holleweg 12, Beek-Ubbergen 6573 DX, The Netherlands

**Keywords:** Self-management, Licensed practical nurses, Aged care, Dual sensory impairment, Hearing impairment, Visual impairment, Cluster randomized controlled trial

## Abstract

**Background:**

Five to 25 percent of residents in aged care settings have a combined hearing and visual sensory impairment. Usual care is generally restricted to single sensory impairment, neglecting the consequences of dual sensory impairment on social participation and autonomy. The aim of this study is to evaluate the effectiveness of a self-management program for seniors who acquired dual sensory impairment at old age.

**Methods/Design:**

In a cluster randomized, single-blind controlled trial, with aged care settings as the unit of randomization, the effectiveness of a self-management program will be compared to usual care. A minimum of 14 and maximum of 20 settings will be randomized to either the intervention cluster or the control cluster, aiming to include a total of 132 seniors with dual sensory impairment. Each senior will be linked to a licensed practical nurse working at the setting. During a five to six month intervention period, nurses at the intervention clusters will be trained in a self-management program to support and empower seniors to use self-management strategies. In two separate diaries, nurses keep track of the interviews with the seniors and their reflections on their own learning process. Nurses of the control clusters offer care as usual. At senior level, the primary outcome is the social participation of the seniors measured using the Hearing Handicap Questionnaire and the Activity Card Sort, and secondary outcomes are mood, autonomy and quality of life. At nurse level, the outcome is job satisfaction. Effectiveness will be evaluated using linear mixed model analysis.

**Discussion:**

The results of this study will provide evidence for the effectiveness of the Self-Management Program for seniors with dual sensory impairment living in aged care settings. The findings are expected to contribute to the knowledge on the program’s potential to enhance social participation and autonomy of the seniors, as well as increasing the job satisfaction of the licensed practical nurses. Furthermore, an extensive process evaluation will take place which will offer insight in the quality and feasibility of the sampling and intervention process. If it is shown to be effective and feasible, this Self-Management Program could be widely disseminated.

**Clinical trials registration:**

ClinicalTrials.gov, NCT01217502.

## Background

As people age, the increasing chance of acquiring a combined hearing and visual impairment adds to the risk of dependency on informal and professional care. In the Dutch population of people aged 80 and over, 5 to 25 percent have a dual sensory impairment (DSI) acquired at old age; the highest percentages were found among seniors living in residential care settings [1].

People with DSI experience barriers in communication, mobility, and information access [2,3], and DSI seniors are at higher risk of depressive feelings [4,5], functional decline [6,7], and social isolation [8]. The consequences of DSI are believed to go beyond those difficulties experienced by seniors suffering from single sensory loss [9], endangering their social participation and autonomy [10,11].

Usual care for seniors with DSI aims to reduce the effects of single sensory impairment, for example by providing technical devices, in some cases supplemented by communication strategies for hearing-impaired seniors, or by training in reading and daily living skills of visually impaired seniors. In some West European countries (for example the Netherlands, UK and Scandinavian countries), home-dwelling seniors with DSI have access to services designed for deafblind adults who acquired dual sensory impairment at an earlier stage in life. These services offer support from social workers and interpreters to address the participation restrictions. In contrast, DSI seniors living in residential care settings are often deprived of special support. Health-care settings are found to be environments where there is limited awareness of the hearing and visual impairment of the patients, leading to a lack of supportive measures in the social and physical environment [12,13]. Hearing or visual impairment is often seen as an individual problem that can be solved by the individual, through medical treatment and technical devices. Sometimes, loneliness and depression are recognized as separate problems, but not as a consequence of a DSI.

Based on what we know from the limited number of available trials, self-management strategies can be expected to be beneficial for sensory-impaired seniors [14], with a potentially higher impact among seniors with depressive feelings [15]. As DSI is associated with participation restrictions, this trial includes social participation as a primary outcome at senior level.

This trial aims to evaluate the effectiveness of a self-management intervention for DSI seniors living in aged care settings. We chose cluster randomization in order to avoid contamination resulting from the effects of possible exchange of information by nurses within the age care setting. We hypothesize that the intervention group will have a more favorable development in social participation, and in nurses’ job satisfaction than the control group. In addition, we hypothesize that seniors suffering from depressive feelings will benefit more from the intervention, and that intervention outcomes at both the level of the seniors and of the nurses will be associated with the adherence of the nurses to the program.

## Methods/Design

### Study design

**Figure 1:**
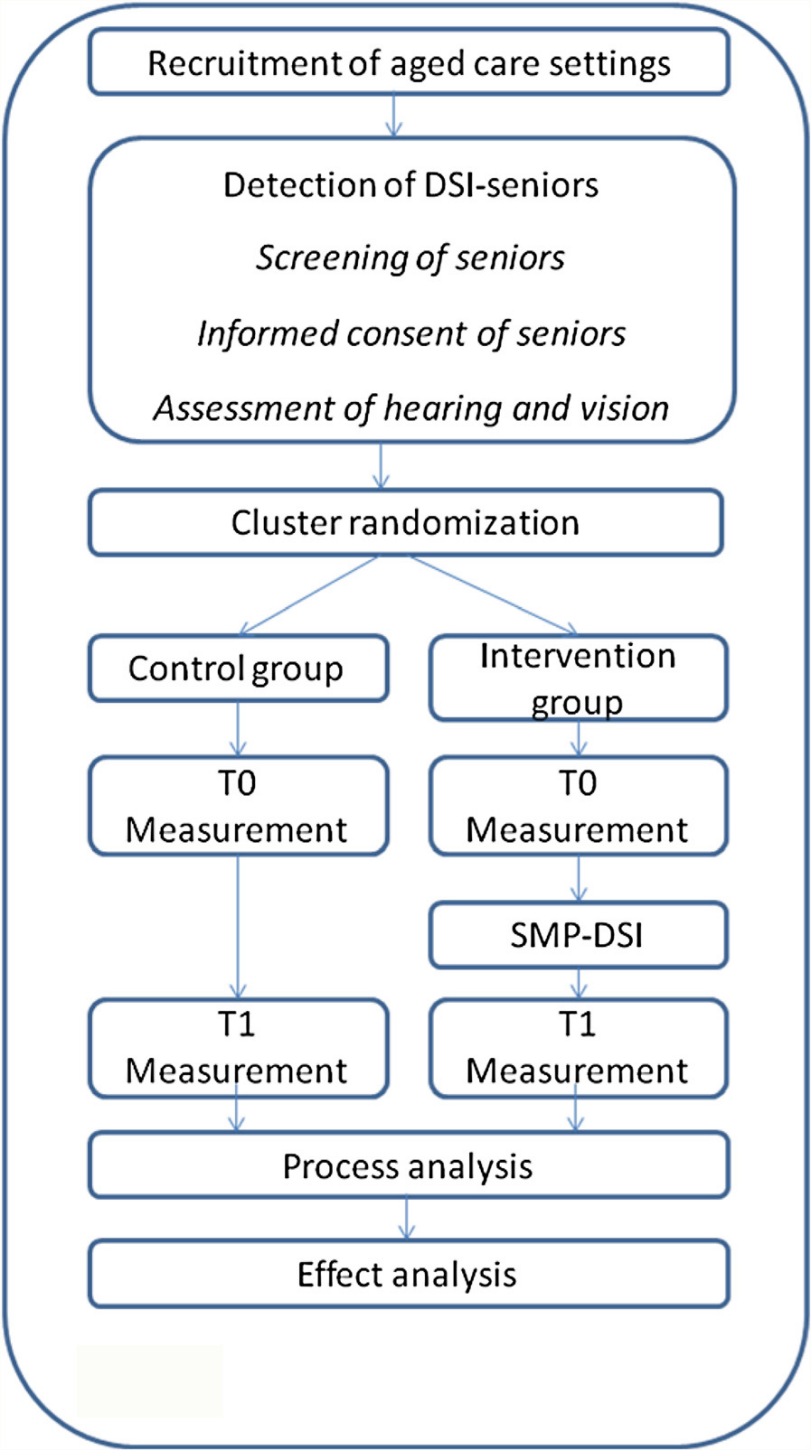
Study design.

The study is designed as a cluster randomized, single-blind controlled trial (Figure 1). A cluster is defined as an aged care setting with an assigned team of nursing staff. A professional caregiver from the setting who has daily care contact with the participating senior will be linked to that senior; we decided to choose licensed practical nurses as they provide the majority of the daily care in aged care settings in the Netherlands. The seniors in the intervention cluster are offered the Self-Management Program for Dual Sensory Impaired Seniors (SMP-DSI) supported by their own nurse. The seniors in the control cluster are offered usual care. An independent statistician will randomize the settings in blocks using a computer-generated random sequence. Data on self-reported outcome measures will be collected at baseline (T0) and four to six weeks after the intervention is finished (T1).

### Study sample

The study sample will consist of age care settings where DSI seniors live. The inclusion criteria for the aged care settings are (1) care organizations offering residential care to seniors, (2) with nursing teams assigned to one setting or location. The inclusion criteria for the care professionals are (1) provision of regular direct daily care (at least twice a week) to the participating senior, (2) qualified as a licensed practical nurse, that is, a three-year basic nursing vocational training at secondary level, and (3) consent given to participate. The inclusion criteria for the seniors are (1) aged 55 or over, (2) a hearing loss of PTA ≥40 dB [16], (3) a visual loss, with a best-corrected visual acuity of <0.3 diopter or with a visual field of <30° [17], and (4) informed consent given by the seniors. Exclusion criteria for the seniors are prelingual deafness, a dual sensory loss acquired before the age of 50, and inability to complete interviews due to cognitive problems.

### Assessment of cognitive functioning in seniors with a dual sensory impairment

To assess cognitive functioning, we will use the DSM IV criteria for capacities in executive functioning: planning, organizing, sequencing and abstracting [18]. These criteria have been selected for their relevance when performing self-management strategies. The list with instructions shows how the DSM IV criteria will be used. We have added these instructions to create valid communication conditions in order to be able to observe cognitive functioning, considering the communication barriers associated with dual sensory impairment.

### List with instructions

DSM IV criteria, capacities for executive functioning:

–planning

–organizing

–sequencing

–abstracting

Step 1. Create valid communication conditions

a. Adapt your output to the auditory and visual needs of the DSI person

Adapt your articulation, face orientation, rhythm and tempo of your speech, and adapt conditions such as lighting, distance, height, and exclude glare and environment noise. If provided, ask the senior to use his/her familiar (hearing) devices

b. If the person does not understand your speech, switch to writing

Adapt size, color and contrast of your writing, adapt paper and pencil type

c. Structure your information

Divide your information into clear parts, avoid sentences with multiple clauses, and pause between each sentence to give the older person time to absorb and comprehend the information

Step 2. Observe cognitive functioning

a. Find proof that the person comprehends your introduction

Is he aware who you are?

Does he comprehend that you want to provide information about a research project?

Does he concentrate on you or your information?

Is he trying to understand and comprehend?

Or does he repeatedly ask who you are, and what you want? Does he persist in talking about his/her own issues or in continuing with own activities?

b. Induce the senior in cognitive planning and reasoning

Ask the persons’ help or preference in planning your next visit; invite him to choose between two or three alternatives (planning)

Invite the person to talk about his experiences with his hearing and vision, and the adaptations he has already established (abstracting, organizing)

Invite the person to tell you what a normal day looks like (sequencing, organizing)

Invite the person to tell you what a weekend day looks like (abstracting, sequencing)

Observe the contribution of the senior during this conversation: are his reactions adequate answers to your questions? Are his answers coherent?

### Procedures

We will start recruitment of the aged care settings by sending an invitational email and an information brochure to the board and the scientific committees of aged care organizations, followed by a personal visit to those organizations interested in participating. Nurses at the participating settings will screen seniors using the Severe Dual Sensory Loss screening tool (SDSL), a questionnaire validated for the Dutch population for DSI [19]. If the SDSL detects DSI-related behavior in a senior, this senior will be invited to participate in the research project, starting with a hearing and vision assessment. Research assistants will observe the cognitive functioning of the senior. Hearing and visual loss will be assessed by a speech therapist and optician. After inclusion of the seniors, licensed practical nurses will be asked to join the program. Each senior will be paired with a nurse; one nurse can be linked to a maximum of two participating seniors.

Cluster randomization occurs after inclusion of seniors in the trial. Settings where no senior participates are therefore not included in the randomization.

### Blinding

The study will be single-blinded, which means that seniors, nurses and trainers will be aware of the allocation arm but will be blinded to the results of any previous assessments. The outcome assessors will be blinded to allocation of the aged care setting of the seniors and the nurses.

### Intervention

#### The self-management program for dual sensory impaired seniors (SMP-DSI)

In this study, the self-management program aims to empower and enable seniors to develop confidence and motivation in the use of their own skills, using resources to participate in a good, safe and emotionally satisfying life in the context of their dual sensory impairment. The SMP-DSI focuses on three self-management tasks to help DSI seniors regain autonomy in their daily lives: (1) to take care of the medical and rehabilitation aspects of the disease (medical management); (2) to carry out normal activities to sustain social participation (role management); and (3) to manage emotional changes as a consequence of being chronically ill (emotional management) [20]. The program has been developed based on the concepts of Bandura’s self-efficacy theory [21], D’Zurilla’s problem-solving theory [22] and Bakker’s constructive behavioral analysis [23]. The structure of the SMP-DSI is a modification of the core self-management skills described by Lorig and Holman combined with the practice-based experiences of social workers and their DSI clients at the Kalorama Foundation [24]. The five steps and related actions of the senior and possible support of the nurses within the program are depicted in Table 1. Due to the communication problems inherent to DSI, the SMP-DSI has been developed using one-to-one interviews. The intervention is delivered at the senior’s residence during daily care contacts, spread over a period of five to six months, starting at the beginning of the nurses’ training program.

**Table 1:** Key features of the self-management program for seniors with dual sensory impairment

**Steps**		**Senior actions**	**Nurse support**		
			**Key questions**	**Ideal support**	**Pitfalls**
**1**	**Problem identification**	Mentions problem. Decides to take action	*You mentioned that you have a problem with…Would you like to do something about it?*	Name the problem using the senior’s own words. If the senior does not want to take action, do not interfere	Ask for an explanation, take on the problem, insistence
**2**	**Collecting alternatives**	Collects a minimum of three alternatives: either by themselves, or by asking others for help	*What could you do about this?*	Stimulate the senior to answer. In cases where the senior does not come up with enough alternatives, provide information. Leave the choice to the senior	Impose solutions, make judgments, provide advice
			*Are there other options?*		
**3**	**Choice and planning**	Selects an alternative that he/she will act on. Plans actions	*How do you think you will manage this?*	If the senior has difficulties planning, then apply the key question 2 procedure	Take over the choice/planning; provide coercive advice
**4**	**Execution**	Executes action		Lets the senior execute the action	Execute action for the senior
**5**	**Reflection**	Reflects on own action, mentions what went well	*What was the result? What are you happy about: about what you could do on your own? What would you do differently the next time?*	Ask What, When, How questions	Ask for an explanation

### Training program for the licensed practical nurses

Nurses in the intervention group will be trained to support the seniors using the SMP-DSI program. The training program consists of nine meetings over a five to six month period, totaling about 18 hours, divided into three rounds; each round consists of a three-hour training session, a 1-hour coaching on the job session, and a one and a half hour supervision session. An interval of two to three weeks is planned between each meeting to give the senior-nurse pairs the opportunity to practice the SMP-DSI in daily care situations.

1. *Training.* The training sessions provide nurses with the background and theory of the SMP-DSI, and focus on the conversational and supporting interview methods needed to assist senior clients in their process of self-management. Each training session involves clearly defined goals and home assignments.

2. *Coaching on the job*. Individual coaching sessions are held to address nurses’ individual experiences regarding the application of the SMP-DSI in practice. Nurses reflect on the process and results of their professional performance.

3. *Supervision.* Participating nurses from different intervention clusters share their experiences using the SMP-DSI and discuss any issues.

Training and supervision sessions are group meetings attended by nurses from one or more settings and led by a trainer. The on-the-job coaching is conducted in individual sessions between the nurse and trainer. Nurses keep two semi-structured diaries: a diary on the interviews with their senior, and a diary with their own learning experiences and consecutive learning goals. Five qualified nurses with long experience of supporting dual sensory impaired seniors will be coached to deliver the SMP-DSI training program to the participating nurses.

### Control condition

Seniors in the control group will receive care as usual, mostly using personal technical devices.

### Outcome measures

At senior level, the primary outcome measure is social participation, as measured by the Hearing Handicap Questionnaire (HHQ) [25,26] and the Activity Card Sort (ACS) [27-29] (see Table 2). The HHQ is a 12-item questionnaire that identifies participation restrictions related to hearing impairment. The ACS has been validated in a number of samples of seniors, and adapted for use by visually impaired seniors. It consists of labeled photographs of older people participating in a range of activities. Completion requires clients to sort the photographs into categories to reflect their current participation. A standardized description of the images of the validated Dutch ACS is read to those participants unable to see the magnified photographs. Secondary outcome measures for the seniors are mood (Centre for Epidemiology Studies Depression Scale (CES-D)) [30], autonomy (Patient Autonomy Questionnaire (PAQ)) [31], perceived control (Pearlin Mastery Scale (PMS)) [32], quality of life (Short-Form 36 Health Survey (SF-36)) [33], and personality-2 factors, extraversion and neuroticism (Neuroticism-Extraversion-Openness Inventory (NEO-FFI)) [34].

**Table 2:** Data sources for measurements and application of variables

**Variable**	**Instrument**	**Application of variable**
**Seniors**		
Social participation	Hearing Handicap Questionnaire (HHQ) [25,26]	Primary outcome
Social participation	Activity Card Sort (ACS) [27-29]	Primary outcome
Mood	Centre for Epidemiology Studies Depression Scale (CES-D) [30]	Secondary outcome
Autonomy	Patient Autonomy Questionnaire (PAQ) [31]	Secondary outcome
Perceived control	Pearlin Mastery Scale (PMS) [32]	Secondary outcome
Quality of life	Short-Form 36 Health Survey (SF-36) [33]	Secondary outcome
Personality-2 factors: Extraversion and Neuroticism	Neuroticism-Extraversion-Openness Inventory (NEO-FFI) [34]	Control variables
Demographic and communication variables		Control variables
**Nurses**		
Job satisfaction	Maastricht Job Satisfaction Scale for Healthcare (MJSS-HC) [35]	Secondary outcome
Demographic variables	Self-developed questionnaire	Control variables
Basic activities of daily living	Katz Index of Independence in Activities of Daily Living [36]	Control variables
Instrumental activities of daily living	Activity Card Sort - subtest IADL (ACS) [27]	Control variables
**Aged care settings**		
Demographic and administrative variables	Self-developed inventory and public sources	Control variables

At nurse level, the outcome measure is job satisfaction, as measured by the Maastricht Job Satisfaction Scale for Healthcare (MJSSH) [35], a questionnaire validated for the Dutch population.

In addition, at senior level we will collect at baseline sociodemographic, personality and communication characteristics. The following variables will be recorded: age, marital status, highest completed education, profession (before retirement), preferred communication modality (speech, written language, tactile language such as hands on or tactile alphabet system), use of hearing or visual devices. Data on functional status will be collected by administering the basic activities of daily living relevant for the target group [36] and the instrumental activities of daily living (ACS-subtest Instrumental activities of daily living (IADL)) score. At nurse level, we will collect data about the educational and professional background of the nurses. At the level of the age care settings, we will collect data on demographic and administrative characteristics.

Two research assistants will collect the data from both seniors and nurses at baseline, and at five to six weeks after the end of the intervention. The research assistants will be blinded for group allocation, and each participant will be followed up by the same assessor. All data from the seniors will be obtained during one-to-one interviews at the senior home environment; data from nurses will be obtained using a written questionnaire. Nurses who meet the participation requirements of 80% will receive a participation certificate from Radboud University Nijmegen and the Kalorama Foundation.

### Sample size and power calculations

For practical reasons, we expect to be able to include 14 to 20 aged care settings, with a maximum of 132 DSI seniors and their nurses. Improvement in either one of the two social participation scales (one scale for hearing-related social participation, and one general scale for social participation), is considered a success; study success is defined as a statistically significant difference on either HHQ or ACS. The enrolment of 14 to 20 settings will enable us to detect an effect size of 0.7 or larger with 80% power on either HHQ or ACS (or equivalently stated, a precision (half-width of the 95% confidence interval for the estimate) of 0.36 standard deviation (SD)), based on the following reasoning: (1) Randomization is at the level of the aged care setting to avoid contamination that may arise should control group nurses come into contact with DSI senior nurses in the intervention group. (2) Uncorrected for clustering, a sample size of 32 per group is needed for the mentioned effect size and power (or equivalently, the stated precision). (3) Given a prevalence of 20% and accounting for low participation rates as found in most studies of very aged and medically compromised seniors, and an average size of a setting (100 to 200 seniors), we expect to recruit five to ten DSI seniors per setting. Typically, a nurse at each setting will coach one to two seniors, so that five nurses from one setting will be involved. Thus, seniors are nested within nurses within aged care settings. To account for this clustering (assuming an intra-nurse correlation between seniors of at most 0.3 and an intra- setting correlation between nurses of at most 0.10), the sample size has to be increased by a factor between 1.12 (if each setting has five nurses each with one senior) and 1.54 (if each setting has five nurses each with two seniors) [37]. In order to allow for an expected 25% dropout, the sample size per group needs to be between 48 and 66, which comes down to between 20 and 14 settings in total. For simplicity of recruitment, we aim to include 132 seniors.

### Statistical methods

Descriptive statistics (mean standard deviation or median and interquartile range) will be used to describe the baseline characteristics of the aged care setting and of the seniors. We will use a linear mixed model to analyze the outcomes and to account for clustering of seniors within nurses within the settings and for repeated measurements. The influence of relevant/prognostic characteristics of aged care setting/seniors on the outcomes will also be investigated by including these as covariates.

The analysis will be based on all the resulting data using the intention-to-treat principle. Moreover, a per protocol analysis and a regression analysis with the adherence to intervention protocol (see below) will be performed to assess the influence of compliance. For all tests, significance will be tested using two-sided tests with an alpha level of .05.

### Process evaluation

In order to assess the internal and external validity of the study, we will evaluate process data on sampling quality (recruitment, randomization and reach) and on intervention quality (relevance, feasibility, adherence and treatment delivery). The data will be performed alongside the SMP-DSI study, and the process evaluation will be executed prior to the effect analysis. Quantitative and qualitative data will be collected from the research database, the intervention diaries and the coaching diaries of the nurses, the semi-standardized records of the trainers, the assessors, the research assistants and the researcher.

To evaluate sampling quality, recruitment and randomization will be defined by description of the recruitment and randomization procedure for the aged care settings; the informed consent and allocation procedure of the seniors; and by description of the barriers and facilitators to the recruiting of the aged care settings, nurses and seniors. Reach will be determined by the proportion of the seniors participating in the SMP-DSI and the number of nurses involved in the intervention.

To evaluate the intervention quality, we will analyze adherence and treatment delivery using data on the frequency and extent to which the SMP-DSI was performed by each pair of senior and nurse, and data on attendance of nurses in the training program. We will also describe the reasons for refusal before the start or during the intervention by aged care setting, nurse and senior. Relevance and feasibility will be defined by the nurses’ diaries and learning goals, the trainers’ training and coaching evaluations, and opinions of the seniors, nurses and trainers about the program. Incentives and barriers toward treatment delivery at the level of aged care settings, nurses and seniors will be classified using the Grol and Wensing framework [38].

### Informed consent and ethical approval

In accordance with the Dutch Medical Research Involving Human Subjects Act (WMO), this study has been approved by the Committee on Research involving Human Subjects of the Arnhem-Nijmegen region, ABR 26192.091.08.

All participating seniors will be requested to sign a consent form prior to data collection.

## Discussion

In this paper we describe the study design for a Self-Management Program for seniors with a Dual Sensory Impairment (SMP-DSI) living in aged care settings, with the aim of improving social participation and autonomy in seniors and job satisfaction in nurses.

This study adds to usual care by expanding the role of the professional caregivers who offer daily care to seniors whose autonomy is threatened. For reasons of feasibility, we only included licensed practical nurses as they are the professional caregivers who offer the majority of the daily care in aged care settings in the Netherlands, but the SMP-DSI could also be used by healthcare assistants or nurse assistants. In the Netherlands, licensed practical nurses and nurse assistants are specially trained in the provision of somatic care and in responding to challenging behavior, but little attention is given to methods aiming at improving seniors’ autonomy and self-management. A strength of this study is that the consecutive coaching sessions offer the professional caregivers solid opportunities to get acquainted with the intervention. However, the long training period increases the risk of drop-out among the aged and vulnerable seniors.

Some specific issues on assessment and outcome measures need to be discussed. First, the assessment of hearing and visual impairment necessary to identify eligible seniors may contribute to the awareness among seniors and nurses of the presence of a DSI, and may influence usual care, resulting in a decrease of the potential effect. Second, we assess cognitive functioning using the DSM IV criteria in relation to the performances needed when executing self-management strategies. Third, we had to tackle a methodological difficulty concerning the selection of outcome measures. As no validated outcome measures on social participation were available for the dual sensory impaired senior population, we selected two outcome measures: one validated for hearing impaired seniors, and one validated in a number of samples of older adults, and adapted for visually impaired seniors. In addition, we included several secondary outcome measures that each captures one or more aspects related to the consequences of a dual sensory impairment, such as mood, autonomy and functional decline (measured by a comprehensive quality of life scale). Moreover, as depressive feelings may impact the results, mood will be considered as an effect modifier for the analysis.

Finally, as recruitment for trials in residential settings and logistic planning is often a challenge, in the research team we will include special members who have access to an extensive network of care providers for seniors, and we will organize the training sessions in the region of the intervention clusters, adopting flexible schedules for the aged care settings. If the SMP-DSI proves effective in the aged care setting, wider implementation will be recommended in order to improve the social participation and autonomy of the seniors.

## Trial status

The trial is currently recruiting aged care settings.

## Competing interests

The authors declare that they have no competing interests. None of the funding agencies had any role in preparing, reviewing or approving the manuscript. They will not be involved in the collection, analysis or interpretation of the data.

## Authors’ contributions

MVD developed the project and obtained funding. LRM is the investigator, has developed the materials for the study, and drafted the manuscript. MVD, GK, MG, SZ and PH contributed to the conception and design of the study, and reviewed and commented on drafts of the manuscript. ST wrote the statistical methods and reviewed drafts of the manuscript. All authors read and approved the final manuscript.
